# Supplementary material for: Complexome profiling of the Chlamydomonas psb28 mutant reveals TEF5 as an early PSII assembly factor
Source: Plant Cell. 2025 Mar 17;37(6):koaf055. doi: 10.1093/plcell/koaf055 (PMC12164590; doi:10.1093/plcell/koaf055)
Supplement: koaf055_Supplementary_Data [file koaf055_supplementary_data.zip › psb28_interactive.zip!/plots/intensity/qualityPlots/peptides/PHO2_Cre07.g336950.t1.1.html]

### Cre07.g336950.t1.1

Trivial name: PHO2  
SeqCoverage [median]: 8.50  
Mass: 98.30 kDa  
  
 

| Sequence | MissedCleavage | Mass [Da] | wtIntensity | muIntensity | Score |
| --- | --- | --- | --- | --- | --- |
| AFASDPAFQAK | 0 | 1151.56 | 0.00 | 593.57 | 113.26 |
| LVTAVGEVINKDPETK | 1 | 1711.94 | 0.00 | 715.81 | 72.17 |
| SIMYTAGSGK | 0 | 1013.49 | 0.00 | 390.63 | 80.79 |
| SSGSAEPVTTDITSK | 0 | 1478.71 | 0.00 | 1160.17 | 142.97 |
| VIAQAYDNPIPGYATPTTSNLR | 0 | 2361.20 | 0.00 | 934.28 | 35.39 |
|  |  |  | 0.0 | 3794.5 |  |
